# Effect of combined G6PD deficiency and diabetes on protein oxidation and lipid peroxidation

**DOI:** 10.1186/s12902-021-00911-6

**Published:** 2021-12-23

**Authors:** Naif S. Karadsheh, Nisreen A. Quttaineh, Salem N. Karadsheh, Mohammad El-Khateeb

**Affiliations:** 1 Department of Biochemistry and Physiology, Faculty of Medicine, The University of Jordan, Amman, Jordan; 2 Faculty of Medicine, The University of Jordan, Amman, Jordan; 3 Department of Internal Medicine, University of Tennessee Graduate School of Medicine, Knoxville, TN USA; 4 The National Center (Institute) for Diabetes, Endocrinology and Genetics, The University of Jordan, Amman, Jordan

**Keywords:** Diabetes, G6PD-deficiency, Oxidative markers, GSH

## Abstract

**Background:**

Oxidative Stress, an imbalance in the pro-oxidant/antioxidant homeostasis, occurs in many physiological and non-physiological processes and several human diseases, including diabetes mellitus (DM) and glucose-6-phosphate dehydrogenase (G6PD) deficiency. Since the incidence of G6PD deficiency in Jordan and many parts of the world is high, this study aimed to measure the effect of G6PD deficiency on the oxidative markers and the antioxidant glutathione (GSH) in diabetic and non-diabetic individuals.

**Methods:**

Whole blood G6PD deficiency was screened by the fluorescent spot method, and erythrocyte G6PD activity was determined using a quantitative assay. Since protein carbonyl (PC) and malondialdehyde (MDA) are the most widely measured markers for protein and lipid oxidation, respectively, plasma PC and MDA, in addition to blood GSH were determined by spectrophotometric assays, as biomarkers of oxidative stress.

**Results:**

The incidence of G6PD deficiency among the diabetic subjects was 15%. PC level in patients with diabetes and in G6PD-deficient subjects was 5.5 to 6-fold higher than in non-diabetic subjects with sufficient G6PD levels (*p*<0.001). This fold increase was doubled in diabetic patients with G6PD deficiency (*p*<0.001). Furthermore, the MDA level was significantly increased by 28-41% in G6PD-deficient, diabetics with sufficient G6PD, and diabetics with G6PD deficiency compared to MDA level in non-diabetic with sufficient G6PD. On the other hand, GSH was significantly reduced to half in G6PD-deficient subjects and in diabetics with G6PD-deficiency.

**Conclusions:**

The results showed that diabetes and G6PD deficiency increased protein oxidation and lipid peroxidation. However, the combination of both disorders has an additive effect only on protein oxidation. On the other hand, GSH level is only reduced in G6PD deficiency. In addition, diabetes and G6PD deficiency appear to be genetically linked since the incidence of G6PD deficiency among people with diabetes is more than the general population.

**Supplementary Information:**

The online version contains supplementary material available at 10.1186/s12902-021-00911-6.

## Background

Glucose-6-phosphate dehydrogenase (G6PD) deficiency is the most common hereditary disorder in humans, found mainly in people of Mediterranean, Southeast Asian, and African descent and affecting over 400 million people [1]. G6PD deficiency is an X-linked disorder caused mainly by diverse point mutations. Although few G6PD variants cause chronic hemolysis, the most common clinical manifestation of its deficiency is neonatal jaundice and oxidative stress-induced hemolytic anemia, caused by certain drugs and chemicals, infections, or ingestion of fava beans (*Vicia faba*) [1, 2].

More than 450 million people worldwide have diabetes mellitus (DM), the vast majority having type 2 DM. Type 2 DM is defined as a group of metabolic disorders characterized by hyperglycemia resulting from defects in action and/or insulin secretion [3]. Hyperglycemia is one of the most important causes of oxidative stress and the production of oxidants. Increased oxidative stress has been suggested to contribute to the pathogenesis and development of diabetic complications [3, 4].

Reactive oxygen species (ROS), including free radicals, are highly reactive molecules generated by the redox reactions that occur as part of normal aerobic cell metabolism and exposure to certain environmental factors [5]. The generation of ROS in cells occurs mainly during energy production. Approximately 2% of total mitochondrial O_2_ consumption result in superoxide production that in turn generates H_2_O_2_ by dismutation [5, 6]. ROS also arise as the product of some enzymatic reactions in the cell. The imbalance between production and scavenging of free radicals due to an increase in the oxidative flux or a decrease in the antioxidant ability is responsible for cellular and tissue damage in several chronic and acute diseases, including DM, cardiovascular disease, cancer, rheumatoid arthritis, Alzheimer’s disease, other neurodegenerative disorders, stroke, sepsis, several hereditary disorders of erythrocytes, and others [7].

The resulting oxidative stress damages cellular components such as proteins, lipids, and nucleic acids [8, 9]. For instance, ROS binding to proteins and lipids modifies amino acid residues generating protein carbonyl (PC) and induces lipid peroxidation generating malondialdehyde (MDA), respectively. Therefore, the quantifications of PC and MDA in the blood are widely used as oxidative biomarkers to evaluate oxidative damage to proteins and lipids [8–10]. Additionally, determining the fate of low molecular antioxidants like reduced glutathione (GSH) is also an important indicator of oxidative damage [10].

In Jordan, the incidence of G6PD deficiency among normal male adults and male newborns is about 5 to 10% [11, 12]. Six different missense mutations were found with a frequency of G6PD Mediterranean about 55% [11]. G6PD deficiency is a public health issue in Jordan because of the hemolytic crisis after ingesting a popular seasonal food, fava beans [11]. In addition, the prevalence of DM (17.1%) and impaired fasting glycemia (7.8%) is high in Jordan and is increasing [13]. The role of G6PD deficiency in the pathogenesis of DM is not certain, but some studies reported that patients who have both disorders showed poorer prognosis [14, 15]. Therefore, the present study aimed to investigate G6PD deficiency’s effect on the levels of oxidative markers and antioxidants in individuals with diabetes and non-diabetics in a population with a high incidence of both.

## Methods

### Subjects and sample processing

Blood samples were collected from diabetic out-patients (*n*=80) at the University of Jordan Hospital and the National Center for Diabetes and from healthy non-diabetic volunteers as controls (*n*=44) that included few known G6PD-deficient subjects. All participants in the study have signed informed consent.

Heparinized blood was collected from the study subjects. A volume of 1.0 ml of whole blood was kept at 4.0℃ and used immediately for GSH estimation and the fluorescent spot screening test for G6PD deficiency [11, 16]. The remaining whole blood was centrifuged at 15,000 rpm for 15 min. Plasma was separated from all the remaining samples and stored at -70℃ until further analysis. The erythrocytes were washed with cold 0.9% saline and stored at -70℃ for G6PD quantification.

### G6PD and GSH assay

The fluorescent spot screening for G6PD deficiency was carried immediately on the blood samples of all participants using whole blood as described by Beutler [11, 16]. Quantitative G6PD assays were performed in all subjects with positive screening tests and 34 subjects with negative screening tests [11, 16]. G6PD levels in G6PD-deficient subjects of both diabetics and non-diabetic were severely deficient with a residual G6PD activity level equal to about 5-10% of the normal level.

GSH was measured in whole blood by the spectrophotometric assay method which involves oxidation of GSH by the sulfhydryl reagent 5,5′-dithio-bis(2-nitrobenzoic acid) (DTNB) to form the yellow derivative 5′-thio-2-nitrobenzoic acid (TNB), measurable at 412 nm [11, 16].

### Determination of plasma MDA and PC

MDA was measured in the plasma calorimetrically using thiobarbituric acid reaction method, as described elsewhere [17]. PC was measured in the plasma by a 2,4dinitrophenyl hydrazine (DPNH) spectrophotometric assay [18].

### Statistical analysis

Data are presented as mean and ± SD. In addition, a comparison between groups was performed by mean of unpaired samples t-test (independent group t-test) for continuous variables using the Statistical Package for Social Science (SPSS version 20). A p value < 0.05 was considered statistically significant.

## Results

The incidence rate of G6PD deficiency among the diabetic subjects (*n*=80) was 15% (12 out of 80) (Table 1), which is higher than our previous reports for the general population and the male newborns in Jordan of 5 to 10% [11, 12]. However, there was no significant difference in the quantitative G6PD activity level between diabetic (*n*=20) and normal subjects (*n*=14) with sufficient-G6PD detected by the negative fluorescent spot screening test (*p* >0.05).

**Table 1:** Level of MDA, PC, and GSH in G6PD-deficient, diabetics with deficient G6PD, diabetics with sufficient G6PD and non-diabetics with sufficient G6PD

Subjects	n	Plasma MDA (nmol/ml)	Plasma PC (nmol/mg protein)	Whole bood GSH (µmol/g Hb)
G6PD-deficient	14	9.33±1.40^*^	3.50±0.95^*^	3.19±1.88^*^
Diabetics with deficient G6PD	12	10.05±0.73^*^	6.53±0.71^*Ϯ^	2.72±0.35^*^
Diabetics with sufficient G6PD	68	9.13±1.88^*^	3.92±0.95^*^	5.85±0.98
Non-Diabetics with sufficient G6PD	30	7.11±1.07	0.64±0.15	6.00±0.40

Values are in mean ±SD ^*^p<0.001 when compared to non-diabetic with sufficient G6PD subjects

^Ϯ^*p*<0.001 when compared to G6PD-deficient and diabetics with sufficient G6PD subjects

MDA, malondialdehyde; PC, protein carbonyl; GSH, reduced glutathione; G6PD, glucose-6- phosphate dehydrogenase

Regarding the oxidative stress status, we observed a 5.5 to 6-fold increase in the PC levels in patients with diabetes and in G6PD-deficient subjects more than in non-diabetic subjects with sufficient G6PD levels (*p*<0.001). This fold increase was doubled in diabetic patients with G6PD deficiency and was significantly higher than in G6PD-deficient subjects and diabetics with sufficient G6PD (*p*<0.001) (Table 1). Furthermore, the MDA level was significantly increased by 28-41% in the G6PD-deficient, diabetics with sufficient G6PD, and diabetics with G6PD deficiency compared to non-diabetic and G6PD-sufficient subjects. On the other hand, GSH was significantly reduced to half in G6PD-deficient subjects and diabetic patients with G6PD-deficiency (*p*<0.001). GSH level in diabetic patients was equivalent to non-diabetic with sufficient G6PD (Table 1).

## Discussion

The balance between ROS and antioxidants determines the oxidative status of cells. Although GSH level is not reduced in diabetic patients since it is likely to be maintained by the NADPH supplied by the normal G6PD, hyperglycemia can promote ROS accumulation through activation of multiple metabolic pathways: (1) increased flux of glucose through the polyol pathway, (2) increased formation of advanced glycation end products (AGEs), (3) activation of protein kinase C (PKC), and (4) increased hexosamine pathway flux [4]. These activated biochemical pathways generate excess ROS leading to oxidative stress and protein and lipid damage, as shown by the nearly 6-fold increase and 28% increase in PC and MDA levels, respectively.

The pentose phosphate pathway is the only source of NAPDH in the erythrocyte. Therefore, G6PD-deficient erythrocytes are unable to maintain GSH to protect sulfhydryl groups against oxidative damage. The role of G6PD deficiency in the pathogenesis of DM is not very clear. Some research suggested no association between them. However, a hypothesis was put that G6PD deficiency could be a risk factor for the occurrence of diabetes [14, 15, 19]. Finally, converging arguments suggest that G6PD deficiency and DM may be etiologically linked [15, 19, 20]. Although our sample is relatively small (80 diabetic patients) but the observed higher incidence of G6PD deficiency among them (15%) than what we reported for the average population (5 -10%) supports the possible genetic connection between these two disorders. In addition, G6PD deficiency should be a disadvantage in patients with diabetes since NADPH is essential to restore the antioxidant GSH. Furthermore, NADPH is a necessary co-factor for the synthesis of NO, a potent vasodilator with anti-atherogenic effects [18, 19]. On the other hand, G6PD deficiency can benefit people with diabetes because decreased NADPH supply may reduce aldose reductase [20]. The latter is the first step in the polyol pathway, which limits the excess of polyols in patients with diabetes and therefore lowers vascular damage, and also accounts for decreased cholesterol synthesis and a favorable lipid profile [18–20].

ROS contribute to the pathogenesis of several hereditary disorders of erythrocytes, including sickle cell anemia (SCA), thalassemia, and G6PD deficiency [6, 10, 19]. Increased oxidative stress in G6PD deficient erythrocytes is documented because of the limited supply of the reducing power NADPH [10]. Therefore, G6PD-deficient erythrocytes are unable to maintain GSH to protect sulfhydryl groups against oxidative damage. Factors responsible for the oxidative stress in thalassemia and SCA are hemoglobin (Hb) instability and excess iron [6, 10]. In thalassemia, excess α- or β-chains are oxidized to metHb and superoxide ion, which is converted to hydroxyl free radical in the presence of released free iron. In SCA, met-HbS is produced at a higher rate, and it is less stable than metHbA, therefore creating more ROS [10]. In thalassemia, the increase in carbonyl groups was 7-fold [10], similar to G6PD deficiency (Table 1). Therefore, the coinheritance of G6PD deficiency and DM is expected to aggravate each other since patients having both disorders have a poorer prognosis [14, 19, 20]. Our data support these clinical findings, as shown by the additive damage in protein oxidation and the decrease in GSH level in DM with G6PD deficiency (Table 1).

Some studies also showed that the clinical impact of G6PD deficiency in patients with SCA could also worsen hemolysis in SCA patients [21, 22], while other studies showed no influence [23]. However, it is imperative to evaluate G6PD status in patients with DM or SCA to avoid drug-induced oxidation. For instance, the hypoglycemic drug, glibenclamide, and the anti-malaria drug, primaquine, both might predispose persons to severe hemolysis [23]. In addition,it will also be interesting to evaluate the association of PC level with the severity of diabetes, especially in G6PD-deficient subjects, and monitor the efficacy of antioxidants supplementation in controlling the PC level.

## Conclusions

The results showed that diabetes and G6PD deficiency increased protein oxidation and lipid peroxidation. However, the combination of both disorders has an additive effect only on protein oxidation. On the other hand, the antioxidant GSH level is only reduced in G6PD deficiency. In addition, diabetes and G6PD deficiency appear to be genetically linked since we observed a higher incidence of G6PD deficiency among people with diabetes than the general population. Therefore, all diabetic patients should be tested for G6PD deficiency to avoid using oxidative drugs that trigger hemolysis and increase complications.

## Supplementary information

**Additional file 1**

## Data Availability

The datasets used and/or analyzed in the current study available from the corresponding author Naif Karadsheh on request.

## Abbreviations

DM: Diabetes mellitus
G6PD: Glucose-6-phosphate dehydrogenase
GSH: Glutathione
PC: Protein carbonyl
MDA: Malondialdehyde
ROS: Reactive oxygen species
SD: Standard deviation
NADPH: Nicotinamide adenine dinucleotide phosphate reduced
SCA: Sickle cell anemia
metHb: methemoglobin
