# Supplementary material for: Effect of combined G6PD deficiency and diabetes on protein oxidation and lipid peroxidation
Source: BMC Endocr Disord. 2021 Dec 23;21:246. doi: 10.1186/s12902-021-00911-6 (PMC8705147; doi:10.1186/s12902-021-00911-6)
Supplement: Supplementary file 1 — Additional file 1 [file 12902_2021_911_MOESM1_ESM.docx]

**References**

1. Nkhoma ET, Poole C, Vannappagari V, Hall SA, Beutler E. The global prevalence of glucose-6-phosphate dehydrogenase deficiency: a systematic review and meta- analysis. Blood Cells Mol Dis. 2009;42:267-78.
2. Luzzatto L. Glucose 6-phosphate dehydrogenase deficiency: from genotype to phenotype. Haematologica. 2006;91:1303-6. PMID: 17018377.
3. Donnely R, Emslie-Smith AM, Gardner ID, Morris AD. ABC of arterial and venous disease: vascular complications of diabetes (Review). BMJ. 2000;320:1062-66.
4. Fiorentino TV, Prioletta A, Zuo P, Foll F. Hyperglycemia-induced oxidative stress and its role in diabetes mellitus related cardiovascular disease. Curr Pharm Des. 2013; 19(32):5695-703.
5. Culler RG. Human Longevity and Aging: Possible role of reactive oxygen species. Ann NY Acad. Sci. 1991;621:1-28.
6. Pereira EC, Ferderbar S, Bertolami MC, Faludi AA, Monte O, Xavier HT, et al. Biomarkers of oxidative stress and endothelial dysfunction in glucose intolerance and diabetes mellitus. Clin Biochem. 2008;41(18):1454-60. doi: 10.1016/j.clinbiochem.2008.08.074. Epub 2008 Sep 3. PMID: 18793627.
7. Bray TM. Antioxidants and oxidative stress in health and disease: Introduction. Proc. Soc. Exp Biol. Med. 1999; 222(3): 195.
8. Dalle-Donne I, Rossi R, Giustarini D, Milzani A, Colombo R. Protein carbonyl groups as biomarkers of oxidative stress. Clinica Chemica Acta. 2003;329:23-8.
9. Niki E, Yoshida Y, Saito Y, Noguchi N. Lipid peroxidation: Mechanisms, inhibition,and biological effects. Biochemical and Biophysical Research Communications. 2005;338:668-76.
10. Fibach E, Rachmilewitz E. The role of oxidative stress in hemolytic anemia. Curr Mol Med. 2008; 8(7):609- 19.

1. Karadsheh NS, Moses L, Ismail SI, Devaney JM, Hoffman E. Molecular heterogeneity of glucose-6-phosphate dehydrogenase deficiency in Jordan. Haematologica, 2005; 90(12):1693-4. PMID: 16330444.
2. .Karadsheh NS, Awidi AS, Tarawneh MC. Two new gucose-6 phosphate dehydrogenase (G6PD) variants associated with hemolytic anemia: G6PD Amman-1 and G6PD Amman-2. Am. J. Hematol. 1986;22:185-92.
3. Ajlouni K, Khader YS, Batieha A, Ajlouni H, El-Khateeb M. An increase in prevalence of diabetes mellitus in Jordan over 10 years. J Diabetes Complicat. 2008; 22: 317-24.
4. Niazi GA. Glucose-6-phosphate dehydrogenase deficiency and diabetes mellitus. Int J Hematol. 1991;54(4):295-8. PMID: 1777604.
5. Santana MS, Monteiro WM, Costa MRF, et al. High frequency of Diabetes and impaired fasting glucose in patients with glucose-6-phosphate dehydrogenase deficiency in the Western Brazilian Amazon. Am J Trop Med Hyg. 2014; 91(1):74-76.
6. Menounos P, Zervas C, Garinis G, Doukas C, Kolokithopoulos D, Tegos C, Patrinos GP. Molecular heterogeneity of the glucose-6-phosphate dehydrogenase deficiency in the Hellenic population. Hum Hered. 2000 ;50(4):237-41. doi: 10.1159/000022922. Erratum in: Hum Hered 2000 ;50(6):369. PMID: 10782016.
7. Nwanjo HU, Oze G, Okafor MC, Nwosu D, Nwankpa P. Protective role of *Phyllantus niruri* extract on serum lipid profiles and oxidative stress in hepatocytes of diabetic rats. Afr J Biotech. 2007; 6:1744- 49.
8. Hernández-Marco R, Codoñer-Franch P, Morales S, Villaescusa CD, García LB, Bellés BG. Oxidant/antioxidant status and hyperfiltration in young patients with type 1 diabetes mellitus. Pediatr Nephrol. 2009; 24:121–27.
9. Lai YK, Lai NM, Lee SW. Glucose-6-phosphate dehydrogenase deficiency and risk of diabetes: a systematic review and meta-analysis. Ann Hematol. 2017; 96 (5): 839-45.
10. Pinna P, Contini EL, Carru C, Solinas G. Glucose-6-phosphate dehydrogenase deficiency and diabetes mellitus with severe retinal complications in a Sardinian population, Italy. Int. J. Med. Sci. 2013; 10:1907-13.

21. Antwi-Baffour S, Adjei JK, Forson PO, Akakpo S, Kyeremeh R, Seidu MA. Comorbidity of glucose-6-Phosphate dehydrogenase deficiency and sickle cell disease exert significant effect on RBC indices. Anemia. 2019; 2019:3179173.

22. Fasola FA, Fowodu FO, Shokunbi WA, Kotila TR. The effect of the coinheritance of glucose-6-phosphate dehydrogenase deficiency on the severity of sickle cell disease. Niger Postgrad Med. 2019; 26(2):118-122.

23. Vinzio S, Andres E, Perrin AE, Schlienger JL, Gaichot B. Glibenclamide-induced acute haemolytic anaemia revealing a G6PD-deficiency. Diabetes Res Clin Pract. 2004; 64:181-183.
